# Exome-Wide Association Analysis Identifies Rare Germline Susceptibility Variants in Early-Onset Breast Cancer Among Saudi Women

**DOI:** 10.3390/ijms27041732

**Published:** 2026-02-11

**Authors:** Rong Bu, Kaleem Iqbal, Sandeep Kumar Parvathareddy, Saud Azam, Zeeshan Qadri, Eman A. Abdul Razzaq, Fouad Al-Dayel, Abdul K. Siraj, Khawla S. Al-Kuraya

**Affiliations:** 1 Genomic Analytics, Research and Innovation, King Faisal Specialist Hospital and Research Centre, Riyadh 11211, Saudi Arabia; 2 Human Cancer Genomic Research, Research and Innovation, King Faisal Specialist Hospital and Research Centre, Riyadh 11211, Saudi Arabia; 3 Translational Oncology Research, Research and Innovation, King Faisal Specialist Hospital and Research Centre, Riyadh 11211, Saudi Arabia; 4 Anatomic Pathology Department, King Faisal Specialist Hospital and Research Centre, Riyadh 11211, Saudi Arabia

**Keywords:** breast neoplasms, young adult, genetic predisposition to disease, germ-line mutation, exome sequencing, Saudi Arabia

## Abstract

Early-onset breast cancer (EOBC) is disproportionately common in Saudi Arabia, where women present nearly a decade earlier than in Western countries, suggesting unique inherited susceptibility. While *BRCA1/2* explain part of the hereditary risk, the contribution of rare coding variants in Arab EOBC remains unclear. Whole-exome sequencing was performed on germline DNA from 102 unrelated Saudi EOBC patients and 1395 cancer-free controls recruited from the same national Saudi cohort. Rare variants were defined by stringent frequency and quality thresholds and classified as rare loss-of-function (RLOF) or rare predicted damaging variants (RPDVs). Gene-level case–control analyses were conducted using burden tests, with exome-wide significance set at *p* < 2.5 × 10^−6^. RLOF variants in *BRCA1* (6.9% of EOBC vs. 0.14% of controls; OR = 51.3; *p* < 1.0 × 10^−10^) and RPDVs in *TP53* (4.9% vs. 0.36%; OR = 14.3; *p* = 5.39 × 10^−8^) demonstrated strong associations. Sequence Kernel Association Test (SKAT) analysis identified *NOTCH4* and *OR12D3* and reinforced burden-based significance in *GUCY2F*, *FRMPD3*, and *SHROOM2*. No enriched signaling pathway emerged, indicating heterogeneous rare-variant mechanisms. This first germline exome-wide rare-variant association study in Saudi EOBC identifies substantial enrichment driven by *BRCA1*, *TP53*, and additional candidate genes, supporting population-specific genetic risk evaluation and the need for replication in larger Arab cohorts.

## 1. Introduction

Breast cancer (BC) is the most commonly diagnosed malignancy among women worldwide [1,2]. A clinically important subset, early-onset breast cancer (EOBC)—defined as diagnosis at ≤50 years—is associated with aggressive tumor biology, reduced survival, and major long-term healthcare impact [3,4,5,6,7,8].

EOBC is particularly frequent in the Middle East. Cancer registry data show that Saudi women are diagnosed with BC nearly 10 years younger than women in Europe or North America, with a significant proportion diagnosed before age 40. According to the Saudi National Cancer Registry, approximately 14–16% of breast cancers in Saudi women are diagnosed before the age of 40, based on age-grouped national incidence data [9,10,11]. EOBC tumors in the region are enriched for high-grade and triple-negative phenotypes [12,13], suggesting an important hereditary contribution beyond demographic structure or reproductive patterns.

Although *BRCA1/2* are primary contributors to hereditary BC [14], pathogenic variants in these genes account for only a minority of EOBC cases. Polygenic common-variant risk scores also explain limited heritability [14,15], indicating additional drivers. Rare, highly penetrant coding variants have been implicated in younger patients with breast cancer [16,17,18,19], and such variants are often population-specific, particularly in regions with high consanguinity, such as Saudi Arabia [17,20].

Despite this, no exome-wide rare-variant case–control study has yet evaluated germline susceptibility in Saudi EOBC patients. This lack of representation in global genomic datasets limits the interpretation of variants and hinders optimal genetic risk management in Arab populations.

To address this gap, we conducted whole-exome-wide association analysis comparing rare loss-of-function and predicted-damaging variants between 102 Saudi EOBC cases and 1395 cancer-free controls recruited from the same national Saudi cohort. Our objectives were to identify susceptibility genes enriched for rare germline variants in EOBC and to generate foundational genomic data to support region-specific genetic testing, risk prediction, and precision-prevention strategies for young women in this understudied population.

## 2. Results

### 2.1. Clinicopathological Characteristics

Median age at diagnosis among the 102 Saudi EOBC patients was 28 years (interquartile range [IQR]: 25.5–30). The predominant histologic subtype was invasive ductal carcinoma (95.1%), with most tumors being moderately or poorly differentiated (93.2%). Stage II disease was most frequent (43.1%), followed by Stage III (23.5%). Triple-negative breast cancer was diagnosed in 23.5% of cases, reflecting the aggressive phenotype characteristic of EOBC in this population (Table 1).

### 2.2. Exome-Wide Rare-Variant Landscape

Across all samples, 263,438 exonic or canonical splice-site variants passed quality filters, including 9968 rare loss-of-function (RLOF) variants (1144 in cases; 9014 in controls) and 143,470 rare predicted damaging variants (RPDVs) (16,944 in cases; 130,874 in controls). The median RLOF burden per individual was similar between cases and controls (10 vs. 9, *p* = 0.510), arguing against a global excess of deleterious variants in EOBC and supporting a model of gene-specific enrichment rather than a generalized increase in rare damaging variation. In contrast, the median RPDV burden was modestly higher in cases compared with controls (135.5 vs. 122; *p* < 0.001), indicating a statistically significant but small increase in overall predicted damaging variation among affected individuals.

### 2.3. High-Penetrance Susceptibility Genes: BRCA1 and TP53

Gene-level association analyses confirmed *BRCA1* and *TP53* as strong susceptibility drivers in Saudi EOBC, as *BRCA1* RLOFs were identified in 7 of 102 EOBC cases (6.9%) and 2 of 1395 controls (0.1%), corresponding to an odds ratio (OR) of 51.32 (*p* < 1.0 × 10^−10^) (Table 2). Whereas *TP53* RPDVs were present in 5 of 102 cases (4.9%) and 5 of 1395 controls (0.4%), yielding an odds ratio of 14.33 (*p* = 5.39 × 10^−8^) (Table 3).

Combined *BRCA1*/*TP53* carrier frequency was 11.8% in cases versus 0.5% in controls, underscoring the substantial contribution of these high-penetrance genes to EOBC risk in this population. Variant-level details, including HGVS notation, population frequencies, and ClinVar annotations, are provided in Supplementary Tables S1 and S2.

### 2.4. X-Linked Rare-Variant Enrichment

A notable concentration of rare predicted damaging variants was observed in several X-linked genes, including *GUCY2F*, *FRMPD3*, *SHROOM2*, *PLXNA3*, *RBMXL3*, *TENM1*, *DMD*, and *GPR112*. All carriers of RPDVs in these genes were EOBC cases, with no corresponding variants in controls. Although the biological relevance of these loci to breast cancer predisposition is not yet established, and mechanistic pathways remain to be elucidated, these genes represent plausible candidate susceptibility loci, particularly in the context of potential sex-specific effects on risk.

These patterns suggest potential high-effect, population-specific contributions to EOBC risk. Full variant counts, annotations, and allele frequencies for candidate genes are reported in Supplementary Table S3.

Due to the inability to perform gender-adjusted modeling, X-linked signals are presented as exploratory and should not be interpreted as evidence of gender-linked susceptibility without gender-adjusted modeling and independent replication.

Several estimates show wide confidence intervals due to low carrier counts; therefore, OR magnitudes, particularly for exploratory and X-linked genes, should be interpreted cautiously.

### 2.5. Candidate EOBC Susceptibility Genes

Beyond known hereditary cancer genes, gene-level SKAT analyses on low-frequency nonsynonymous variants identified five additional candidate EOBC susceptibility genes achieving exome-wide significance (Table 4), three of which overlapped with burden-based exome-wide significant genes (*GUCY2F*, *FRMPD3*, *SHROOM2*). Variant-level details have been provided in Supplementary Table S4.

### 2.6. Pathway-Level Findings

To evaluate whether associated genes converged on shared biological processes, KEGG pathway enrichment analysis was performed using the set of genes harboring rare predicted damaging variants. KEGG pathway enrichment analysis did not identify any pathways that remained significant after FDR correction (FDR ≥ 0.05). This lack of pathway-level convergence is consistent with allelic heterogeneity, modest cohort size, and the likelihood that EOBC susceptibility in this population involves diverse molecular mechanisms rather than a single dominant signaling axis.

## 3. Discussion

Next-generation sequencing (NGS) has expanded understanding of cancer genetics, yet a large proportion of early-onset breast cancer (EOBC) heritability remains unaccounted for, particularly in underrepresented populations. This knowledge gap is pronounced in the Arab region, where breast cancer is diagnosed at significantly younger ages and where comprehensive germline investigations are limited. Here, we leveraged whole-exome sequencing (WES) to assess the contribution of rare coding variants to EOBC predisposition in Saudi women and to identify candidate susceptibility genes warranting further study.

Using harmonized sequencing and bioinformatic pipelines across 102 EOBC cases and 1395 cancer-free controls recruited from the same national Saudi cohort, we applied stringent frequency and pathogenicity filters to prioritize rare loss-of-function and predicted-damaging variants. This strategy enabled robust gene-level association analysis while minimizing technical and population stratification artifacts.

Consistent with established biology, *BRCA1* demonstrated the strongest association signal, with a ~51-fold case enrichment of rare deleterious variants. This supports *BRCA1* as a dominant hereditary contributor to EOBC in Saudi women and reinforces its priority in genetic testing and counseling protocols. Detection of two pathogenic *BRCA1* variants in controls likely reflects limited cohort size, incomplete longitudinal follow-up, or age-dependent penetrance, underscoring the need for future family-based segregation and clinical annotation.

*TP53* was the second most significant gene (~14-fold enrichment), aligning with its recognized role in Li–Fraumeni spectrum cancers and very-early-onset breast cancer. Together, *BRCA1* and *TP53* accounted for 11.8% of EOBC cases, emphasizing substantial high-penetrance contribution in this population.

The enrichment of rare deleterious variants in *BRCA1* and *TP53* observed in this study is consistent with their established role as high-penetrance susceptibility genes for EOBC reported in Western and Asian populations. Previous exome-based rare-variant studies have similarly demonstrated a disproportionate contribution of *BRCA1* and *TP53* to EOBC, although reported carrier frequencies vary substantially across populations. Importantly, to our knowledge, no prior exome-wide rare-variant association study has specifically evaluated germline susceptibility in EOBC among Saudi or Arab populations. Our findings therefore provide the first population-specific exome-wide evidence supporting both shared high-penetrance drivers and potential ancestry-linked differences in the genetic architecture of EOBC.

Beyond known hereditary genes, we identified multiple case-exclusive rare variants in genes achieving exome-wide significance, including *GUCY2F*, *FRMPD3*, and *SHROOM2*. Replicated significance across burden and SKAT models provides convergent evidence within this dataset. However, this does not constitute replication, and independent validation is required. Their biological relevance to breast cancer development remains to be defined; however, they represent a focused and novel set of candidates for functional validation and replication in independent EOBC cohorts.

An additional observation was the clustering of rare-variant signals on the X chromosome, including *DMD*, *GPR112*, *GUCY2F*, *FRMPD3*, *PLXNA3*, *RBMXL3*, *SHROOM2*, and *TENM1*, with all carrier status restricted to EOBC cases. These concentrations of signals on the X chromosome should be interpreted cautiously. Rare-variant aggregation on sex chromosomes can be influenced by gene size, coverage heterogeneity, and technical calling differences; furthermore, biological interpretation is complicated by X-inactivation and dosage effects in females. Accordingly, X-linked candidate genes identified here should be considered hypothesis-generating and require replication and functional validation.

Pathway-level analysis did not reveal statistically significant enrichment. The observed results likely reflect allelic heterogeneity and modest cohort size. This suggests that EOBC risk in this population is distributed across diverse biological mechanisms, rather than being centralized in established pathways.

Because this is a case–control study, odds ratios reflect enrichment of variant carriers among cases versus controls and should not be interpreted as population-level absolute risk or penetrance.

Key strengths of this study include being the first exome-wide rare-variant association analysis of EOBC in an Arab population.

Limitations include limited statistical power to detect moderate-effect variants, absence of longitudinal outcome data for controls, and lack of functional validation or segregation studies. In addition, a modest sample size for rare-variant discovery could result in wide uncertainty for genes with low carrier counts. ORs may be unstable under sparse data and should be interpreted as enrichment rather than penetrance. Where confidence intervals span orders of magnitude, effect-size estimates are unstable under sparse data and are presented primarily as descriptive enrichment rather than precise magnitude. Age distribution was unavailable for the full control cohort, and age adjustment was not feasible; therefore, some controls may not yet have passed through the age window at risk for EOBC, potentially inflating the observed enrichment among cases. X-chromosome analyses are subject to technical and biological complexities. Therefore, X-chromosome association results should be interpreted with caution. The unavailability of gender information for a subset of controls precluded gender-adjusted modeling and formal inference for X-linked genes. Furthermore, penetrance and absolute risk cannot be inferred from this design and require population-based longitudinal cohorts. Moreover, recruitment from a single institution may not fully capture the genetic diversity of the broader Saudi or Arab populations. Due to access limitations, raw sequencing files and genome-wide genotype data for the control cohort were not available, precluding formal assessment of sequencing quality-control metrics, batch effects, or ancestry inference using principal component analysis. Since genome-wide markers and PCA-based ancestry inference were not available, residual population stratification cannot be fully excluded

Despite these constraints, our results demonstrate that EOBC in Saudi women is driven not only by high-penetrance genes (*BRCA1*, *TP53*) but also by a broader spectrum of rare variation, including novel and X-linked candidate susceptibility loci. These findings provide essential baseline data for an understudied population and support expansion of multigene panel testing and future precision-prevention strategies tailored to young Arab women at elevated risk of breast cancer.

## 4. Materials and Methods

### 4.1. Study Population

We analyzed germline whole-exome sequencing (WES) data from 102 unrelated Saudi female patients diagnosed with EOBC (≤50 years) at King Faisal Specialist Hospital and Research Center (KFSHRC), Riyadh, between 2000 and 2020. Clinico-pathological features—including age, tumor histology, grade, TNM stage, and receptor status—were abstracted from institutional medical records. All samples were collected prior to systemic therapy. Ethical approval was granted by the KFSHRC Institutional Review Board (RAC# 2140008), with waiver of informed consent for archival anonymized samples.

As a control group, we included 1395 cancer-free individuals recruited from the same national Saudi cohort. These individuals were enrolled based on diverse Mendelian traits unrelated to cancer, all of whom had available exome sequencing data. All genomic analyses for cases and controls were performed using the same sequencing platform, bioinformatic processing, and variant filtering pipeline to minimize technical bias.

### 4.2. DNA Extraction and Whole-Exome Sequencing

Genomic DNA was extracted from peripheral blood or non-neoplastic tissue using the Gentra Puregene DNA Isolation Kit (Qiagen, Germantown, MD, USA), following the manufacturer’s protocol. Exome enrichment was performed using Agilent SureSelect XT capture kits (Agilent Technologies, Santa Clara, CA, USA) followed by paired-end sequencing on Illumina HiSeq systems.

### 4.3. Alignment, Variant Calling, and Quality Control

Sequencing reads were aligned to the human reference genome (hg19) using BWA-MEM. PCR duplicates were removed using Picard. Local indel realignment and base-quality recalibration were performed using the Genome Analysis Toolkit (GATK v4.0.12.0). Variant calling for all samples (cases and controls) was conducted jointly using GATK HaplotypeCaller to generate a cohort VCF, ensuring consistent detection and genotyping across groups.

Rare variants were retained if they met all of the following criteria: depth ≥ 10×, genotype quality ≥ 20, exonic or canonical splice-site location, and a minor allele frequency (MAF) < 0.01 in gnomAD, ExAC, and internal Saudi population data. Variant annotation was performed using ANNOVAR (version 20200608).

### 4.4. Variant Classification

Rare Loss-of-Function (RLOF) variants included frameshift, stop-gain/loss, and essential splice-site variants. Rare Predicted Damaging Variants (RPDVs) were defined as rare missense variants with high deleteriousness scores (CADD Phred ≥ 20 and/or M-CAP ≥ 0.025). Exome-wide association analyses were performed using both RLOF and RPDVs. Low-frequency nonsynonymous variants (MAF < 0.1) were additionally analyzed using SKAT.

### 4.5. Statistical Analysis

Sequence Kernel Association (SKAT) analyses were conducted in R (version 4.1.2) using the SKAT package (version 2.2.5), whereas other statistical tests were performed in SPSS (Version 22). Gene-based case–control association testing was performed for genes harboring ≥5 rare variants in cases. Burden testing was performed using a two-sided χ^2^ test or Fisher’s exact test, as appropriate, and the SKAT was used to assess aggregated effects of low-frequency nonsynonymous variants (MAF < 0.1). Exome-wide significance was defined using Bonferroni correction for approximately 20,000 genes (0.05/20,000 ≈ 2.5 × 10^−6^). Odds ratios (ORs) and 95% confidence intervals (CIs) were calculated for significant gene-level findings using Firth penalized logistic regression to address complete separation due to zero-cell counts.

### 4.6. Pathway Enrichment Analysis

Functional enrichment of genes harboring rare predicted damaging variants was evaluated using Kyoto Encyclopedia of Genes and Genomes (KEGG) pathway analysis implemented in DAVID v6.8 with multiple-testing correction by FDR (FDR < 0.05).

## Figures and Tables

**Table 1:** Clinico-pathological variables for the patient cohort (*n* = 102).

Variable	*n* (%)
**Age**	
Median (in years)	28.0
Range (IQR)	25.5–30.0
**Histological type**	
Infiltrating Ductal Carcinoma	97 (95.1)
Infiltrating Lobular Carcinoma	2 (2.0)
Mucinous Carcinoma	2 (2.0)
Unknown	1 (0.9)
**Tumor laterality**	
Right	57 (55.9)
Left	42 (41.2)
Bilateral	2 (2.0)
Unknown	1 (0.9)
**Family history of cancer**	
Negative	81 (79.4)
Positive	19 (18.6)
Unknown	2 (2.0)
**TNM Stage**	
I	17 (16.7)
II	44 (43.1)
III	24 (23.5)
IV	11 (10.8)
Unknown	6 (5.9)
**Histologic Grade**	
Well Differentiated	1 (0.9)
Moderately Differentiated	42 (41.2)
Poorly Differentiated	53 (52.0)
Unknown	6 (5.9)
**Estrogen Receptor**	
Positive	59 (57.8)
Negative	38 (37.3)
Unknown	5 (4.9)
**Progesterone Receptor**	
Positive	46 (45.1)
Negative	51 (50.0)
Unknown	5 (4.9)
**Her-2 neu**	
Positive	28 (27.5)
Negative	69 (67.6)
Unknown	5 (4.9)
**Triple Negative Breast Cancer**	
Yes	24 (23.5)
No	73 (71.6)
Unknown	5 (4.9)

**Table 2:** Genes with exome-wide significant RLOFs in our cohort.

S. No.	Gene	No. of Cases	% Cases	No. of Controls	% Controls	*p*-Value	Odds Ratio (95% CI)
1	*BRCA1*	7	6.9	2	0.1	<1.0 × 10^−10^	51.3 (10.5–250.5)

OR and 95% CI are derived from gene-level carrier status using Firth logistic regression.

**Table 3:** List of genes with significant RPDVs at the exome-wide level (*p* < 2.5 × 10^−6^).

S. No.	Gene	Chr	No. of Cases	% Cases	No. of Controls	% Controls	*p*-Value	Odds Ratio (95% CI)
1	*KCNH2*	chr7	6	5.9	7	0.5	1.57 × 10^−8^	12.39 (4.1–37.6)
2	*CCHCR1*	chr6	5	4.9	0	0.0	<1.0 × 10^−10^	157.4 (17.7–20,724)
3	*MARCO*	chr2	5	4.9	7	0.5	1.50 × 10^−6^	10.22 (3.2–32.8)
4	*PODNL1*	chr19	5	4.9	7	0.5	1.50 × 10^−6^	10.22 (3.2–32.8)
5	*TP53*	chr17	5	4.9	5	0.4	5.39 × 10^−8^	14.33 (4.1–50.3)
6	*PACS2*	chr14	7	6.9	13	0.9	4.75 × 10^−7^	7.83 (3.1–20.1)

OR and 95% CI are derived from gene-level carrier status using Firth logistic regression.

**Table 4:** List of genes significant at the exome-wide level analyzed by SKAT.

S. No.	Gene	No. of Cases	% Cases	No. of Controls	% Controls	*p*-Value	Odds Ratio (95% CI)
1	*NOTCH4*	5	4.9	0	0.0	6.69 × 10^−7^	157.4 (17.7–20,724.0)
2	*GUCY2F*	6	5.9	0	0.0	4.35 × 10^−8^	188.0 (22.0–24,584.7)
3	*FRMPD3*	5	4.9	0	0.0	6.69 × 10^−7^	157.4 (17.7–20,724.0)
4	*SHROOM2*	6	5.9	0	0.0	4.35 × 10^−8^	188.0 (22.0–24,584.7)
5	*OR12D3*	6	5.9	0	0.0	4.35 × 10^−8^	188.0 (22.0–24,584.7)

ORs (95% CI) are derived from gene-level carrier-status logistic regression (Firth) and are descriptive only; statistical significance for this table is based on SKAT gene-based *p*-values.

## Data Availability

The original contributions presented in the study are included in the article/Supplementary Materials; further inquiries can be directed to the corresponding author/s.

## Supplementary Materials

The following supporting information can be downloaded at: https://www.mdpi.com/article/10.3390/ijms27041732/s1.
