# Supplementary material for: Exome-Wide Association Analysis Identifies Rare Germline Susceptibility Variants in Early-Onset Breast Cancer Among Saudi Women
Source: Int J Mol Sci. 2026 Feb 11;27(4):1732. doi: 10.3390/ijms27041732 (PMC12940663; doi:10.3390/ijms27041732)
Supplement: Supplementary file 1 [file ijms-27-01732-s001.zip › ijms-4074002-supplementary-updated.pdf]

Supplementary Table S1: List of rare loss-of-function variants associated with EOBC risk.

S No	Gene	Chr	Position (hg19)	HGVS c.	HGVS p.	Variant Type	Case Count	Control Count	gnomAD MAF	ClinVar Classification
1	*BRCA1*	chr17	41197757	c.5530delC	p.L1844Sfs*11	frameshift deletion	1	0	NA	Pathogenic
2	*BRCA1*	chr17	41199696	c.C5431T:	p.Q1811X	stopgain	1	0	NA	Pathogenic
3	*BRCA1*	chr17	41209095	c.C1939T	p.R647X	stopgain	1	0	0.00003228	Pathogenic
4	*BRCA1*	chr17	41215890	c.5152+1G>C	-	splicing	1	0	NA	Pathogenic
5	*BRCA1*	chr17	41226499	c.G4524A	p.W1508X	stopgain	1	0	NA	Pathogenic
6	*BRCA1*	chr17	41244585	c.C2963A	p.S988X	stopgain	1	0	NA	Pathogenic
7	*BRCA1*	chr17	41246407	c.1140dupG	p.K381Efs*3	frameshift insertion	1	0	NA	Pathogenic
8	*BRCA1*	chr17	41246169	c.1378dupA	p.I460Nfs*20	frameshift insertion	0	1	NA	Pathogenic
9	*BRCA1*	chr17	41251897	c.C442T	p.Q148X	stopgain	0	1	NA	VUS

Supplementary Table S2: List of rare predicted damaging variants associations with EOBC risk.

S No	Gene	Chr	Position (hg19)	HGVS c.	HGVS p.	Variant Type	Case Count	Control Count	gnomAD MAF	CADD (Phred)	M-CAP Score	ClinVar
1.	*TP53*	chr17	7573988	c.G922A	p.A308T	missense	1	0	NA	27.00	0.422	VUS
2.	*TP53*	chr17	7574002	c.G908C	p.R303P	missense	1	0	NA	16.35	0.728	Pathogenic
3.	*TP53*	chr17	7577094	c.C448T	p.R150W	missense	1	0	NA	20.80	0.268	Pathogenic/Likely pathogenic
4.	*TP53*	chr17	7577121	c.C421T	p.R141C	missense	1	0	0	17.48	0.265	Pathogenic/Likely pathogenic
5.	*TP53*	chr17	7578508	c.G26A	p.C9Y	missense	1	0	NA	15.06	0.24	Pathogenic/Likely pathogenic
6.	*TP53*	chr17	7579906	c.G7A	p.E3K	missense	0	1	NA	17.47	0.485	VUS
7.	*TP53*	chr17	7579479	c.G208T	p.A70S	missense	0	1	NA	5.63	0.822	NA
8.	*TP53*	chr17	7577069	c.G473A	p.R158H	missense	0	1	0.0001	8.23	0.842	Benign
9.	*TP53*	chr17	7579482	c.G205C	p.A69P	missense	0	1	NA	12.65	0.383	VUS
10.	*TP53*	chr17	7577151	c.A391G	p.N131D	missense	0	1	NA	13.49	0.347	VUS
11.	*TENM1*	chrX	123514646	c.G7918A	p.E2640K	missense	1	0	0.00004631	27.80	0.143	NA
12.	*TENM1*	chrX	123515036	c.G7528A	p.G2510R	missense	1	0	0.0001	9.09	0.304	Likely benign
13.	*TENM1*	chrX	123517498	c.C7262A	p.A2421E	missense	1	0	NA	8.84	0.41	NA
14.	*TENM1*	chrX	123556388	c.G4184A	p.R1395H	missense	1	0	NA	22.5	0.257	VUS
15.	*TENM1*	chrX	124029931	c.G377C	p.R126T	missense	1	0	NA	15.49	0.51	NA
16.	*SHROOM2*	chrX	9862768	c.G820A	p.G274S	missense	1	0	0.0002	7.86	0.999	Likely benign
17.	*SHROOM2*	chrX	9863047	c.G1099A	p.D367N	missense	1	0	NA	17.54	0.602	NA
18.	*SHROOM2*	chrX	9863497	c.C1549T	p.R517C	missense	1	0	0.0014	12.05	0.783	Benign
19.	*SHROOM2*	chrX	9863548	c.C1600T	p.R534W	missense	1	0	0.00004691	9.75	0.991	VUS
20.	*SHROOM2*	chrX	9864413	c.C2465T	p.P822L	missense	1	0	0.00009178	1.22	0.998	Likely benign
21.	*SHROOM2*	chrX	9900318	c.C2995T	p.R999W	missense	1	0	NA	10.86	0.998	NA
22.	*SHROOM2*	chrX	9907258	c.C668T	p.A223V	missense	1	0	0.00004639	9.60	0.601	Likely benign
23.	*RBMXL3*	chrX	114424860	c.G856T	p.G286C	missense	1	0	0.0028	12.59	0.999	NA
24.	*RBMXL3*	chrX	114424933	c.C929T	p.P310L	missense	1	0	0.0008	9.77	1	NA
25.	*RBMXL3*	chrX	114424953	c.T949C	p.W317R	missense	1	0	0.0028	0.003	0.999	NA
26.	*RBMXL3*	chrX	114425680	c.C1676G	p.S559W	missense	1	0	0.0026	0.64	0.999	NA

S No	Gene	Chr	Position (hg19)	HGVS c.	HGVS p.	Variant Type	Case Count	Control Count	gnomAD MAF	CADD (Phred)	M-CAP Score	ClinVar
27.	*RBMXL3*	chrX	114426012	c.G2008A	p.E670K	missense	1	0	0.00005054	9.94	1	VUS
28.	*RBMXL3*	chrX	114426241	c.G2237A	p.R746H	missense	1	0	0.0007	8.52	0.998	VUS
29.	*RBMXL3*	chrX	114426378	c.G2374A	p.G792R	missense	1	0	0	8.87	0.999	NA
30.	*RBMXL3*	chrX	114426612	c.G2608A	p.D870N	missense	1	0	NA	14.35	1	NA
31.	*RBMXL3*	chrX	114426886	c.G2882A	p.S961N	missense	1	0	0.0026	6.77	1	NA
32.	*PODNL1*	chr19	14043618	c.C1193T	p.A398V	missense	1	0	NA	13.29	0.764	NA
33.	*PODNL1*	chr19	14043868	c.G943A	p.A315T	missense	1	0	0.00003235	17.53	0.733	NA
34.	*PODNL1*	chr19	14043981	c.G830A	p.R277H	missense	1	2	0.0007	15.64	0.844	NA
35.	*PODNL1*	chr19	14044039	c.C772A	p.H258N	missense	1	0	NA	21.80	0.909	NA
36.	*PODNL1*	chr19	14044066	c.G745A	p.G249R	missense	1	0	0.0005	11.20	0.827	VUS
37.	*PODNL1*	chr19	14045177	c.C316T	p.P106S	missense	1	0	NA	5.09	0.998	NA
38.	*PODNL1*	chr19	14048774	c.G64A	p.V22I	missense	0	2	0.0004	12.93	0.833	NA
39.	*PODNL1*	chr19	14044056	c.G980A	p.R327Q	missense	0	1	NA	8.53	0.921	VUS
40.	*PODNL1*	chr19	14047232	c.C107T	p.A36V	missense	0	1	NA	11.55	0.99	NA
41.	*PODNL1*	chr19	14044047	c.G989A	p.R330Q	missense	0	1	0.00006475	22.80	0.89	NA
42.	*PLXNA3*	chrX	153688713	c.C190T	p.R64W	missense	1	0	0.0002	19.81	0.827	Likely benign
43.	*PLXNA3*	chrX	153689488	c.C644T	p.T215M	missense	1	0	NA	19.92	0.599	NA
44.	*PLXNA3*	chrX	153692331	c.C1585T	p.P529S	missense	1	0	NA	16.09	0.749	NA
45.	*PLXNA3*	chrX	153692792	c.G1876A	p.V626M	missense	1	0	0.00009292	8.52	0.838	NA
46.	*PLXNA3*	chrX	153695621	c.G3248A	p.G1083D	missense	1	0	NA	22.30	0.558	NA
47.	*PLXNA3*	chrX	153696456	c.G3852T	p.E1284D	missense	1	0	NA	7.78	0.868	NA
48.	*PACS2*	chr14	105834470	c.G646A	p.A216T	missense	1	0	NA	21.00	0.871	Likely benign
49.	*PACS2*	chr14	105847373	c.C1115T	p.T372M	missense	1	0	0.0005	7.38	0.959	Benign/Likely benign
50.	*PACS2*	chr14	105848854	c.T1352A	p.L451H	missense	1	0	NA	19.32	0.718	VUS
51.	*PACS2*	chr14	105849811	c.G1639A	p.D547N	missense	1	0	0.0005	18.01	0.881	Likely benign
52.	*PACS2*	chr14	105859018	c.G1639A	p.D547N	missense	1	3	0.00003234	6.85	0.983	Benign/Likely benign
53.	*PACS2*	chr14	105859594	c.G1639A	p.D547N	missense	1	0	NA	9.33	0.966	NA
54.	*PACS2*	chr14	105860986	c.G1639A	p.D547N	missense	1	0	NA	18.29	0.789	VUS
55.	*PACS2*	chr14	105814894	c.G184A	p.V62M	missense	0	1	NA	15.11	0.876	Likely benign

S No	Gene	Chr	Position (hg19)	HGVS c.	HGVS p.	Variant Type	Case Count	Control Count	gnomAD MAF	CADD (Phred)	M-CAP Score	ClinVar
56.	*PACS2*	chr14	105859135	c.C2300T	p.T767M	missense	0	1	0.00003234	17.00	0.918	Benign/Likely benign
57.	*PACS2*	chr14	105833650	c.C524T	p.S175F	missense	0	1	NA	18.43	0.867	NA
58.	*PACS2*	chr14	105833710	c.C584T	p.T195M	missense	0	1	NA	10.98	0.935	VUS
59.	*PACS2*	chr14	105848366	c.G1384A	p.A462T	missense	0	1	NA	0.25	0.974	Benign
60.	*PACS2*	chr14	105848862	c.G1450A	p.D484N	missense	0	1	NA	16.75	0.899	VUS
61.	*PACS2*	chr14	105850741	c.A1820G	p.N607S	missense	0	1	NA	3.05	0.988	Benign
62.	*PACS2*	chr14	105851249	c.C1913T	p.T638M	missense	0	1	0.00006476	8.16	0.952	Likely benign
63.	*PACS2*	chr14	105849820	c.G1648A	p.G550S	missense	0	1	NA	16.37	0.99	VUS
64.	*PACS2*	chr14	105847306	c.C1048T	p.P350S	missense	0	1	0.0001	9.76	0.995	VUS
65.	*MARCO*	chr2	119729104	c.G454A	p.A152T	missense	1	0	0.00006462	1.22	0.849	VUS
66.	*MARCO*	chr2	119731998	c.G550C	p.G184R	missense	1	1	NA	5.41	0.672	NA
67.	*MARCO*	chr2	119732001	c.C553G	p.R185G	missense	1	0	NA	5.16	0.803	NA
68.	*MARCO*	chr2	119735053	c.A620C	p.Q207P	missense	1	0	NA	7.20	0.752	NA
69.	*MARCO*	chr2	119739771	c.C941T	p.A314V	missense	1	0	NA	12.18	0.998	NA
70.	*MARCO*	chr2	119749410	c.C1166A	p.A389D	missense	0	2	NA	13.55	0.964	NA
71.	*MARCO*	chr2	119748168	c.T1067C	p.L356P	missense	0	1	NA	12.98	0.922	NA
72.	*MARCO*	chr2	119735089	c.C656G	p.T219S	missense	0	1	NA	7.06	0.938	NA
73.	*MARCO*	chr2	119727884	c.C394A	p.Q132K	missense	0	1	NA	9.18	0.835	NA
74.	*MARCO*	chr2	119750717	c.G1270A	p.V424I	missense	0	1	0.0002	10.16	0.988	VUS
75.	*KIAA1817*	chrX	106844589	c.C3320T	p.A1107V	missense	1	0	0.00004691	7.58	0.984	NA
76.	*KIAA1817*	chrX	106844918	c.C3649T	p.R1217C	missense	1	0	0.0005	15.69	0.882	NA
77.	*KIAA1817*	chrX	106845375	c.G4106A	p.G1369D	missense	1	0	NA	6.15	0.962	NA
78.	*KIAA1817*	chrX	106845408	c.G4139A	p.G1380D	missense	1	0	NA	13.92	0.947	NA
79.	*KIAA1817*	chrX	106845732	c.G4463A	p.S1488N	missense	1	0	NA	7.17	0.991	NA
80.	*KIAA1817*	chrX	106846524	c.C5255G	p.T1752R	missense	1	0	NA	5.51	0.984	NA
81.	*KCNH2*	chr7	150644759	c.C1880T	p.P627L	missense	1	0	0.0003	8.68	0.472	Benign/Likely benign
82.	*KCNH2*	chr7	150645995	c.G1521T	p.E507D	missense	1	2	NA	14.31	0.567	VUS
83.	*KCNH2*	chr7	150655312	c.C751T	p.P251S	missense	1	0	NA	10.59	0.071	VUS
84.	*KCNH2*	chr7	150655366	c.G697T	p.A233S	missense	1	0	NA	9.39	0.098	Benign/Likely benign

S No	Gene	Chr	Position (hg19)	HGVS c.	HGVS p.	Variant Type	Case Count	Control Count	gnomAD MAF	CADD (Phred)	M-CAP Score	ClinVar
85.	*KCNH2*	chr7	150655425	c.A638G	p.D213G	missense	1	0	NA	11.87	0.068	VUS
86.	*KCNH2*	chr7	150655521	c.G542A	p.R181Q	missense	1	0	0.0025	14.37	0.435	Benign/Likely benign
87.	*KCNH2*	chr7	150648074	c.C1060T	p.R354C	missense	0	1	NA	18.75	0.125	VUS
88.	*KCNH2*	chr7	150656690	c.C442T	p.R148W	missense	0	1	0.0005	17.43	0.229	VUS
89.	*KCNH2*	chr7	150649787	c.C263T	p.S88L	missense	0	1	0.00003232	19.01	0.218	VUS
90.	*KCNH2*	chr7	150649611	c.G439C	p.G147R	missense	0	1	NA	16.92	0.541	NA
91.	*KCNH2*	chr7	150644066	c.G2209A	p.A737T	missense	0	1	NA	27.60	0.738	VUS
92.	*GUCY2F*	chrX	108619377	c.C3170G	p.T1057S	missense	1	0	0.00009225	16.35	0.442	NA
93.	*GUCY2F*	chrX	108638614	c.G2380A	p.E794K	missense	1	0	0.0043	27.40	0.798	Likely benign
94.	*GUCY2F*	chrX	108641880	c.A2173G	p.R725G	missense	1	0	NA	8.87	0.876	NA
95.	*GUCY2F*	chrX	108647605	c.G2077A	p.D693N	missense	1	0	NA	13.04	0.833	NA
96.	*GUCY2F*	chrX	108696974	c.A1147G	p.R383G	missense	1	0	0.0002	9.52	0.881	NA
97.	*GUCY2F*	chrX	108718645	c.G521A	p.R174Q	missense	1	0	0.0000926	9.43	0.408	NA
98.	*GPR112*	chrX	135429475	c.A3610T	p.T1204S	missense	1	0	0.0034	7.31	0.985	NA
99.	*GPR112*	chrX	135430342	c.A4477G	p.N1493D	missense	1	0	NA	14.05	0.974	VUS
100.	*GPR112*	chrX	135430504	c.T4639A	p.C1547S	missense	1	0	0.0013	10.62	0.982	Likely benign
101.	*GPR112*	chrX	135432554	c.C6689T	p.T2230M	missense	1	0	NA	0.02	0.988	NA
102.	*GPR112*	chrX	135441599	c.G7129A	p.A2377T	missense	1	0	NA	11.61	0.68	NA
103.	*DMD*	chrX	31227768	c.A206G	p.N69S	missense	1	0	NA	13.70	0.889	Benign
104.	*DMD*	chrX	31792244	c.C3343G	p.L1115V	missense	1	0	NA	8.76	0.934	VUS
105.	*DMD*	chrX	32430156	c.C19T	p.R7X	stopgain	1	0	NA	16.13	.	NA
106.	*DMD*	chrX	32472949	c.G3433C	p.V1145L	missense	1	0	NA	14.00	0.377	Benign/Likely benign
107.	*DMD*	chrX	32663135	c.A1071C	p.Q357H	missense	1	0	0.0003	18.91	0.957	VUS
108.	*CCHCR1*	chr6	31125191	c.187delA	p.S63Afs*7	frameshift deletion	1	0	0.0006	.	.	NA
109.	*CCHCR1*	chr6	31112526	c.G1679A	p.R560Q	missense	1	0	0.0007	16.15	0.898	NA
110.	*CCHCR1*	chr6	31118527	c.G809A	p.R270Q	missense	1	0	0.00009696	11.09	0.979	VUS
111.	*CCHCR1*	chr6	31118875	c.G559A	p.E187K	missense	1	0	NA	13.42	0.978	NA
112.	*CCHCR1*	chr6	31124836	c.G247C	p.E83Q	missense	1	0	0.0003	19.72	0.988	NA

Supplementary Table S3: Exploratory X-linked findings (unadjusted)

S No	Gene	Chr	No. of Cases	% Cases	No. of Controls	% Controls	p-value	Odds ratio (95% CI)
1	*TENM1*	chrX	5	4.9	0	0.0	$<1.0 \times 10^{-10}$	157.4 (17.7-20724.0)
2	*GUCY2F*	chrX	6	5.9	0	0.0	$<1.0 \times 10^{-10}$	188.0 (22.0-24584.7)
3	*PLXNA3*	chrX	6	5.9	0	0.0	$<1.0 \times 10^{-10}$	188.0 (22.0-24584.7)
4	*FRMPD3*	chrX	6	5.9	0	0.0	$<1.0 \times 10^{-10}$	188.0 (22.0-24584.7)
5	*SHROOM2*	chrX	7	6.9	0	0.0	$<1.0 \times 10^{-10}$	219.19 (26.4-28525.9)
6	*RBMXL3*	chrX	5	4.9	0	0.0	$<1.0 \times 10^{-10}$	157.4 (17.7-20724)
7	*DMD*	chrX	5	4.9	0	0.0	$<1.0 \times 10^{-10}$	157.4 (17.7-20724)
8	*GPR112*	chrX	5	4.9	0	0.0	$<1.0 \times 10^{-10}$	157.4 (17.7-20724)

OR and 95% CI are derived from gene-level carrier status Firth logistic regression

Supplementary Table S4: List of variants significantly related to EOBC risk in five SKAT genes.

S No	Gene	Chr	Position	HGVS c.	HGVS p.	Variant Type	Case Count	Control Count	gnomAD MAF	CADD (Phred)	ClinVar
1	*SHROOM2*	chrX	9862768	c.G820A	p.G274S	missense	1	0	0.0002	7.87	Likely benign
2	*SHROOM2*	chrX	9863047	c.G1099A	p.D367N	missense	1	0	NA	17.54	NA
3	*SHROOM2*	chrX	9863548	c.C1600T	p.R534W	missense	1	0	0.00004691	9.76	VUS
4	*SHROOM2*	chrX	9864413	c.C2465T	p.P822L	missense	1	0	0.00009178	1.22	Likely benign
5	*SHROOM2*	chrX	9900318	c.C2995T	p.R999W	missense	1	0	NA	10.86	NA
6	*SHROOM2*	chrX	9907258	c.C668T	p.A223V	missense	1	0	0.00004639	9.60	Likely benign
7	*OR12D3*	chr6	29342359	c.G706T	p.A236S	missense	2	0	0.0031	17.02	NA
8	*OR12D3*	chr6	29342832	c.A233G	p.K78R	missense	2	0	0.00006458	11.13	NA
9	*OR12D3*	chr6	29342653	c.G412T	p.V138L	missense	1	0	0.0000323	0.05	VUS
10	*OR12D3*	chr6	29342767	c.C298T	p.H100Y	missense	1	0	0.00003228	0.005	NA
11	*NOTCH4*	chr6	32188865	c.G689A	p.R230H	missense	2	0	0.0003	1.16	NA
12	*NOTCH4*	chr6	32170009	c.G3599A	p.G1200E	missense	1	0	0.0003	13.53	NA
13	*NOTCH4*	chr6	32170264	c.C3344A	p.A1115D	missense	1	0	0.0007	15.29	NA
14	*NOTCH4*	chr6	32189084	c.G470C	p.R157P	missense	1	0	NA	12.23	NA
15	*GUCY2F*	chrX	108619377	c.C3170G	p.T1057S	missense	1	0	0.00009225	16.35	NA
16	*GUCY2F*	chrX	108638614	c.G2380A	p.E794K	missense	1	0	0.0043	27.40	Likely benign
17	*GUCY2F*	chrX	108641880	c.A2173G	p.R725G	missense	1	0	NA	8.87	NA
18	*GUCY2F*	chrX	108647605	c.G2077A	p.D693N	missense	1	0	NA	13.04	NA
19	*GUCY2F*	chrX	108696974	c.A1147G	p.R383G	missense	1	0	0.0002	9.52	NA
20	*GUCY2F*	chrX	108718645	c.G521A	p.R174Q	missense	1	0	0.0000926	9.43	NA
21	*FRMPD3*	chrX	106844589	c.C3320T	p.A1107V	missense	1	0	0.00004691	7.58	NA
22	*FRMPD3*	chrX	106844918	c.C3649T	p.R1217C	missense	1	0	0.0005	15.69	NA
23	*FRMPD3*	chrX	106845408	c.G4139A	p.G1380D	missense	1	0	NA	13.92	NA
24	*FRMPD3*	chrX	106845732	c.G4463A	p.S1488N	missense	1	0	NA	7.17	NA
25	*FRMPD3*	chrX	106846524	c.C5255G	p.T1752R	missense	1	0	NA	5.51	NA
